# Pluronic P123 modified nano micelles loaded with doxorubicin enhanced tumor-suppressing effect on drug-resistant breast cancer cells

**DOI:** 10.18632/aging.103138

**Published:** 2020-05-12

**Authors:** Xiaoyu Zhang, Weibin Chen, Jie Bai, Lijun Jin, Xiaoning Kang, Hui Zhang, Zunyi Wang

**Affiliations:** 1 Department of Thyroid and Breast III, Cangzhou Central Hospital, Cangzhou, Hebei Province, China; 2 Department of Radiology, North China University of Science and Technology Affiliated Hospital, Tangshan, Hebei Province, China; 3 Department of Ultrasound II, Cangzhou Central Hospital, Cangzhou, Hebei Province, China

**Keywords:** nano micelles, multi-drug resistance, breast cancer, P-glycoprotein, Pluronic P123 modification

## Abstract

Objective: Nano micelles (NMs) have been widely used for various biomedical applications due to its unique physiochemical properties. This study aimed to investigated the anti-tumor effect of doxorubicin (Dox)-loaded Pluronic P123 (P123) and PEG2000-DSPE mixed NMs in drug-resistant breast cancer cells.

Results: The expression of P-gp and MDR1 gene was highly expressed in MCF-7R but not MCF-7 cells. The cellular uptake of P123-PEG2000-DSPE (Dox) was higher than that of free Dox and PEG2000-DSPE (Dox) in MCF-7R cells. Furthermore, compared with free Dox, both PEG2000-DSPE (Dox) and P123-PEG2000-DSPE (Dox) significantly diminished cell viability, and promoted cell apoptosis in MCF-7R cells. In addition, the P123-modified NMs obviously inhibited the expression of P-gp and MDR1.

Conclusions: P123-PEG2000-DSPE (Dox) had a superior anti-tumor activity than PEG2000-DSPE (Dox) in MCF-7R cells through P-gp-mediated drug excretion and drug resistance mechanisms.

Methods: The PEG2000-DSPE NMs (PEG2000-DSPE), P123 and PEG2000-DSPE mixed NMs (P123-PEG2000-DSPE), Dox-loaded PEG2000-DSPE NMs (PEG2000-DSPE (Dox)), and Dox-loaded Pluronic P123 and PEG2000-DSPE mixed NMs (P123-PEG2000-DSPE (Dox)) were prepared, and then the morphologies and the size distribution of PEG2000-DSPE (Dox) and P123-PEG2000-DSPE (Dox) were observed by transmission electron microscopy (TEM) and dynamic light scattering (DLS), respectively.

## INTRODUCTION

As the common malignant tumors, breast cancer (BC) usually originates from the mammary epithelial tissues, with high rates of morbidity and mortality [1, 2]. Nowadays, surgical resection, chemotherapy, and targeted pharmacological treatments are the conventional therapeutic measures for BC [3–5]. Although there has been intensive progress in the diagnosis and treatments for BC, unsatisfactory prognosis and poor survival still exist [6]. Especially, one of the major obstacles for cancer treatment is drug resistance to chemotherapeutic drugs, which should be responsible for the failed chemotherapy [7]. Thus, it is essential to reveal the underlying drug resistance mechanisms, as well as search for novel treatment methods for BC.

Currently, cumulative researched have focused on MDR mechanisms in cancer, and several proteins have been proved to be related to MDR, such as MDR protein, BC resistance protein, P-glycoprotein (P-gp), and lung resistance related protein [8, 9]. Importantly, P-gp, an ATP-binding cassette transporter, is thoroughly studied as the key mechanism of multi-drug resistance (MDR) in cancers [10, 11]. It has been reported that P-gp encoded by *MDR1* gene plays pivotal role in the efflux mechanism, which can pump to extrude various kinds of drugs out of MDR cancer cells acting as a drug efflux [10, 11]. Thus, inhibiting P-gp expression is considered as a possible strategy for the resensitization of MDR cancer cells through mediating drugs efflux, thereby improving the success rate of chemotherapy in patients with MDR tumor [12–14].

Doxorubicin (Dox) is a common antitumor drug that can inhibit the synthesis of RNA and DNA [15]. However, the clinical use of Dox is compromised for efficient cancer treatment due to its low bioavailability, and other side effects caused by nonspecific cytotoxicity, as well as MDR cancer cells [16]. Thus, it is indispensable to design an effective targeted drug delivery system that not only is able to increase the concentration of drugs in tumor cells, but also resensitize to MDR cancer cells. Currently, nano-micelles (NMs) are reported to be a promising drug delivery system, and widely applied in cancer therapy [17, 18]. As the most common nanocarriers, NMs possess several favorable properties such as high biocompatibility and ability to carry large drug payloads [17]. Previous studies have developed Dox-loaded NMs, and Dox-loaded NMs exhibits improved tumor-suppressing effects compared with free Dox [19, 20]. Notably, Pluronic P123 (P123), a common type of Pluronic copolymer, is reported to exhibit prominent toxicity to MDR cells by suppressing the expression of P-gp [21]. Several studies have demonstrated that P123 modified nanocarrier has potential to improve the treatment of MDR tumors by inhibiting the expression of P-gp [22–24]. Therefore, in the current research, we coupled P123 with PEG2000-DSPE in Dox-loaded NMs, and then characterization and *in vitro* drug release of NMs were detected. In addition, the effects of NMs on cell proliferation and apoptosis in MCF-7 and MDR MCF-7R cells were explored.

## RESULTS

### Characterization and in vitro drug release of NMs

**Figure 1:**
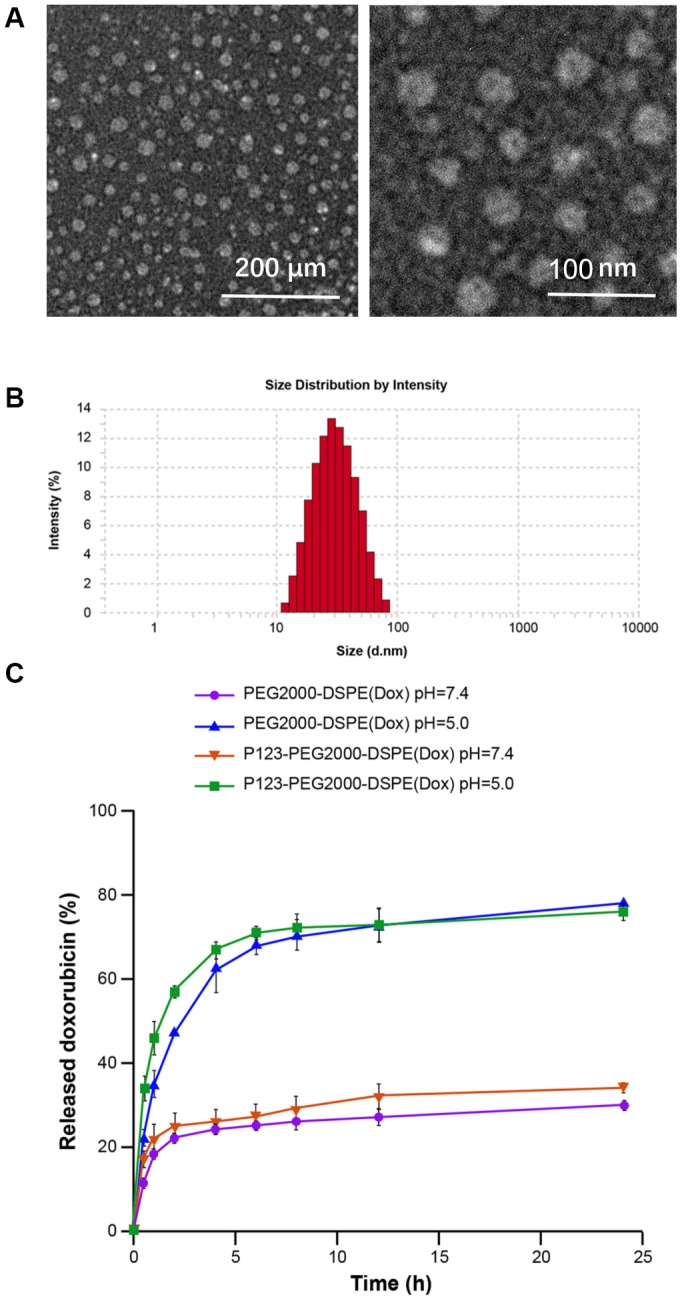
**Characterization of P123-PEG2000-DSPE (Dox).** (**A**) The morphological characteristics of P123-PEG2000-DSPE (Dox) observed by transmission electron microscope. (**B**) Size distributions of P123-PEG2000-DSPE (Dox) determined by dynamic light scattering. (**C**) Cumulative drug release profiling of PEG2000-DSPE (Dox) and P123-PEG2000-DSPE (Dox) in phosphate-buffer saline (PBS, pH 7.4 and pH 5.0).

As shown in Figure 1A, TEM showed spherical shape of P123-PEG2000-DSPE (Dox) with the size of ~50 nm (Figure 1A). Accordingly, DLS revealed that the mean hydrodynamic diameter of P123-PEG2000-DSPE (Dox) was observed to be around 50 nm (Figure 1B), and the parameter chart is shown in Table 1. As shown in Table 2, the LC, EE and zeta potential of P123-PEG2000-DSPE (Dox) were 16.8%, 99.4% and -4.9 ± 1.4 (mV), respectively. Next, the amount of Dox released from PEG2000-DSPE (Dox) and P123-PEG2000-DSPE (Dox) was examined at pH 7.4 and pH 5.0. Cumulative drug release profiles revealed that both PEG2000-DSPE (Dox) and P123-PEG2000-DSPE (Dox) exhibited a burst release of Dox within 5 h and a slow release from 5 h to 24 h at pH 7.4 and pH 5.0 (Figure 1C). Notably, almost 80 % of Dox was released from both PEG2000-DSPE (Dox) and P123-PEG2000-DSPE (Dox) within 24 h at pH 5.0, which was significantly higher than that at pH 7.4 (about 30%) (Figure 1C).

**Table 1:** The parameter chart of P123-PEG2000-DSPE (Dox) by dynamic light scattering.

**Parameter**	**10-20 nm**	**20-30 nm**	**30-40 nm**	**40-50 nm**	**50-60 nm**	**60-70 nm**	**70-80 nm**	**80-90 nm**	**90-100 nm**
Size distribution (%)	2.1	6.3	14.2	21.8	23.1	15.2	10.3	5.8	1.2

**Table 2:** Physicochemical properties of P123-PEG2000-DSPE (Dox).

**Zeta potential (mV)**	**Drug loading capacity**	**Encapsulation efficiency**
-4.9 ± 1.4	16.8%	99.4%

### Expression of P-gp in MCF-7 and MCF-7R cells

**Figure 2:**
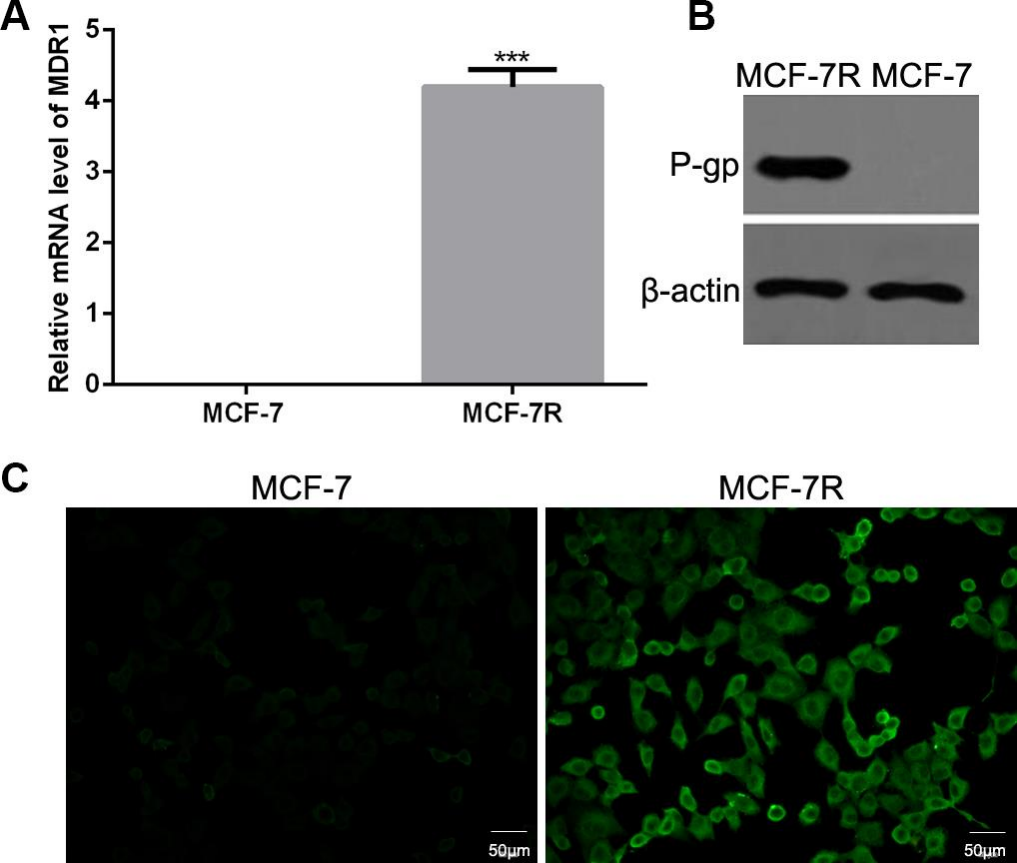
**Expression of P-glycoprotein (P-gp) in MCF-7 and MCF-7R cells.** (**A**) The mRNA levels of multi-drug resistance (MDR1) gene in MCF-7 and MCF-7R cells by qRT-PCR. (**B**) The protein expression of P-gp in MCF-7 and MCF-7R cells using western blotting. (**C**) The expression of P-gp in MCF-7 and MCF-7R cells by cell immunofluorescence.

The mRNA and protein expression levels of P-gp were detected in MCF-7 and MCF-7R cells. The results of qRT-PCR showed that the mRNA level of MDR1 was highly expressed in MCF-7R cells but not MCF-7 cells (Figure 2A). Consistently, both western blotting (Figure 2B) and immunofluorescence (Figure 2C) revealed higher expression level of P-gp in MCF-7R cells than that in MCF-7 cells.

### Cellular uptake of NMs in vitro

**Figure 3:**
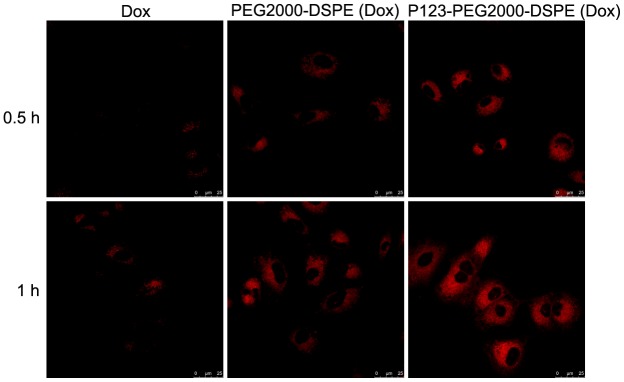
**Cellular uptake of Dox, PEG2000-DSPE (Dox) or P123-PEG2000-DSPE (Dox) by MCF-7R cells.** Confocal images of MCF-7R cells treated with Dox, PEG2000-DSPE (Dox) or P123-PEG2000-DSPE (Dox) at 10 μg/mL for 0.5 and 1 h.

Based on confocal imaging analysis, we found the enhanced fluorescence intensity when cells were treated with PEG2000-DSPE (Dox) or P123-PEG2000-DSPE (Dox) compared with free Dox, especially, the strongest fluorescence intensity was found in cells treated with P123-PEG2000-DSPE (Dox) (Figure 3). These results indicated that the addition of P123 increased the cellular uptake of NMs. Moreover, the cellular uptake at 1 h of incubation time was higher than 0.5 h (Figure 3).

### In vitro anti-proliferation effect of NMs

**Figure 4:**
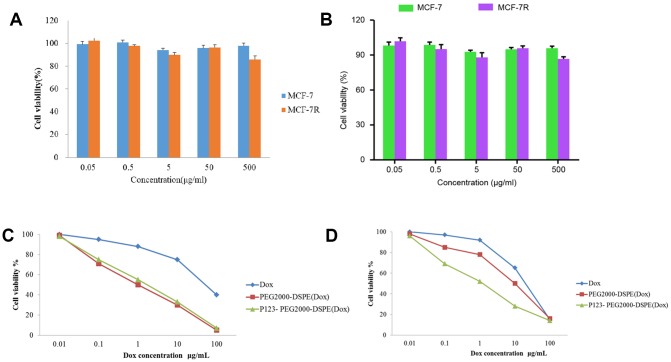
**P123-PEG2000-DSPE (Dox) inhibited cell growth in MCF-7 and MCF-7R cells.** (**A**) Cell viability of MCF-7 and MCF-7R cells treated with different doses of PEG2000-DSPE (unloaded nano micelles) at 24 h by MTT assay. (**B**) Cell viability of MCF-7 and MCF-7R cells treated with different doses of P123-PEG2000-DSPE at 24 h by MTT assay. (**C**) Cell viability of MCF-7 cells treated with PBS (control), Dox, PEG2000-DSPE (Dox) or P123-PEG2000-DSPE (Dox) by MTT assay. (**D**) Cell viability of MCF-7R cells treated with PBS (control), Dox, PEG2000-DSPE (Dox) or P123-PEG2000-DSPE (Dox) by MTT assay.

Cell proliferation and apoptosis was used to evaluate the antitumor effect of GPC1-LP (GEM). Firstly, the cytotoxicity of PEG2000-DSPE (unloaded NMs) and P123-PEG2000-DSPE was evaluated by MTT assay, and the results found that different concentrations of PEG2000-DSPE or P123-PEG2000-DSPE treatments exhibited little toxicity to both MCF-7 and MCF-7R cells (Figure 4A, 4B). In addition, the results found that MCF-7 cells treated with PEG2000-DSPE (Dox) exhibited similar cell viability with MCF-7 cells with P123-PEG2000-DSPE (Dox), while both which were reduced compared with cells with free Dox (Figure 4C). Compared with MCF-7R cells treated with free Dox, cell viability was decreased in MCF-7R cells with PEG2000-DSPE (Dox), and cells treated with P123-PEG2000-DSPE (Dox) showed lower cell viability than that in cells with PEG2000-DSPE (Dox) (Figure 4D).

### Effect of NMs on P-gp expression in vitro

**Figure 5:**
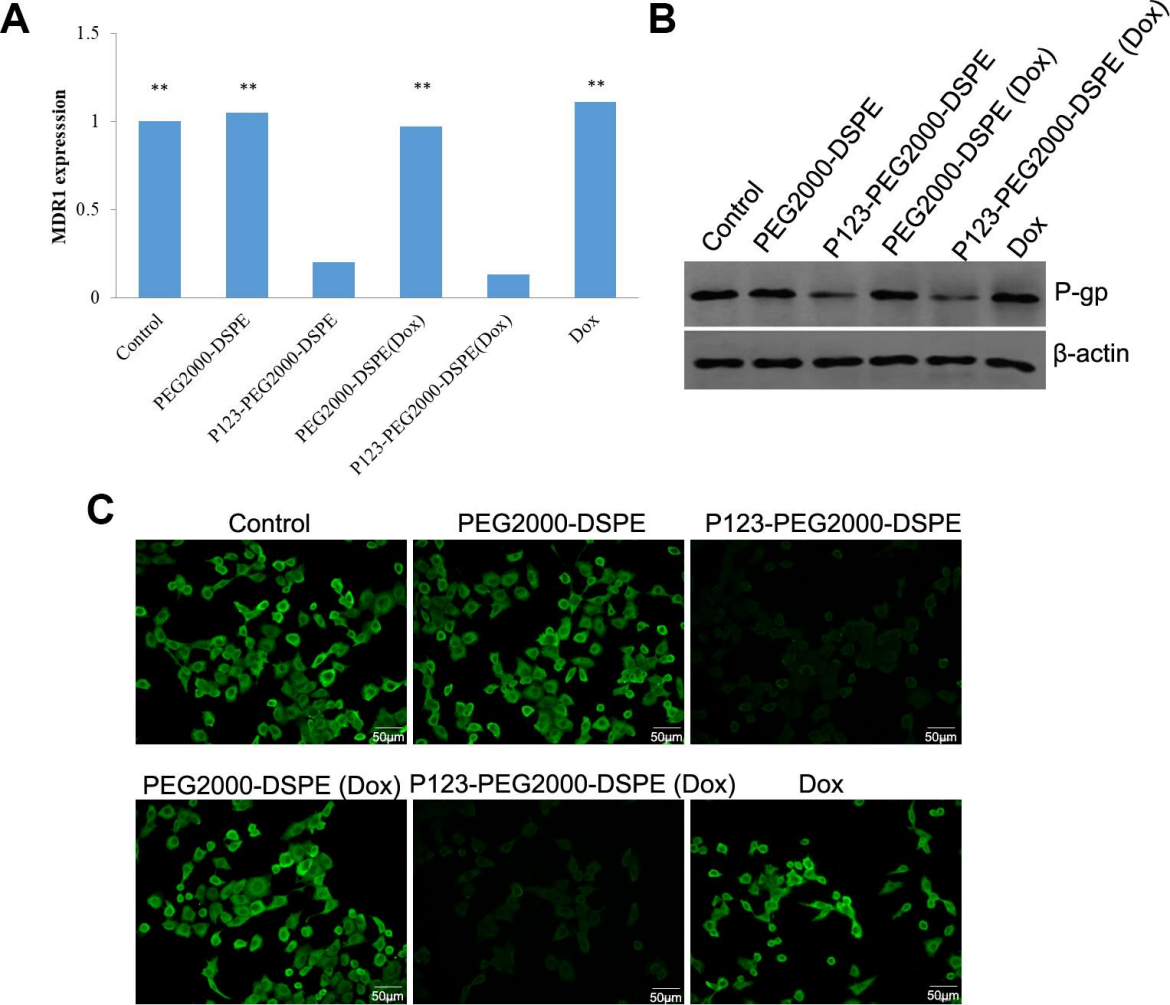
**P123-PEG2000-DSPE (Dox) and P123-PEG2000-DSPE inhibited the expression of P-glycoprotein (P-gp) in MCF-7R cells.** (**A**) The mRNA levels of multi-drug resistance (MDR1) gene in MCF-7R cells treated with PBS (control), PEG2000-DSPE, P123-PEG2000-DSPE, Dox, PEG2000-DSPE (Dox) and P123-PEG2000-DSPE (Dox) by qRT-PCR. (**B**) The protein expression of P-gp in MCF-7R cells underwent various treatments using western blotting. (**C**) The expression of P-gp in MCF-7R cells underwent various treatments by cell immunofluorescence.

MCF-7R cells were treated with PBS (control), PEG2000-DSPE, P123-PEG2000-DSPE, Dox, PEG2000-DSPE (Dox) and P123-PEG2000-DSPE (Dox), respectively, and then the mRNA and protein expression levels of P-gp were detected. The results of qRT-PCR found similar mRNA levels of MDR1 in control cells as well as cells treated with PEG2000-DSPE, free Dox and PEG2000-DSPE (Dox) (Figure 5A). However, compared these cells, the mRNA level of MDR1 was significantly decreased in cells with P123-PEG2000-DSPE and P123-PEG2000-DSPE (Dox), especially in cells with P123-PEG2000-DSPE (Dox) (Figure 5A). Consistently, both western blotting (Figure 5B) and immunofluorescence (Figure 5C) revealed similar expression trend of P-gp protein when MCF-7R cells underwent the above treatments.

### In vitro pro-apoptosis effect of NMs

**Figure 6:**
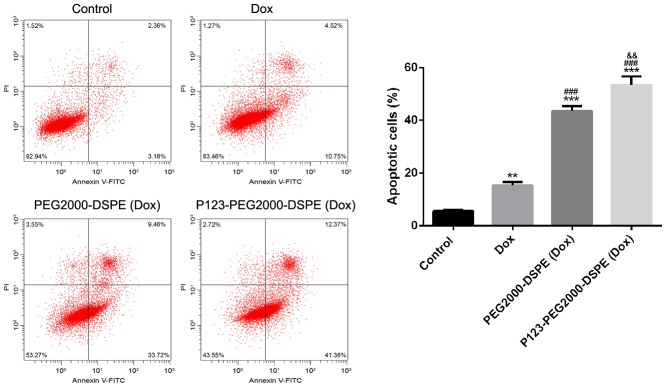
**P123-PEG2000-DSPE (Dox) induced cell apoptosis in MCF-7R cells.** Cell apoptosis rate of MCF-7R cells treated with PBS (control), Dox, PEG2000-DSPE (Dox) or P123-PEG2000-DSPE (Dox) by flow cytometry analysis. **P < 0.01 and ***P < 0.001 vs. control group; ^###^P < 0.001 vs. Dox group; ^&&^P < 0.01 vs. PEG2000-DSPE (Dox) group.

Flow cytometry analysis revealed that the free Dox significantly increased the rate of apoptotic cells compared with control cells; meanwhile, compared with cells with free Dox, cells treated with PEG2000-DSPE (Dox) showed the increased rate of apoptotic cells, and then P123-PEG2000-DSPE (Dox) treatment further increased the rate of apoptotic cells (p < 0.01, Figure 6).

### Effect of NMs on drug-resistant BC mice

**Figure 7:**
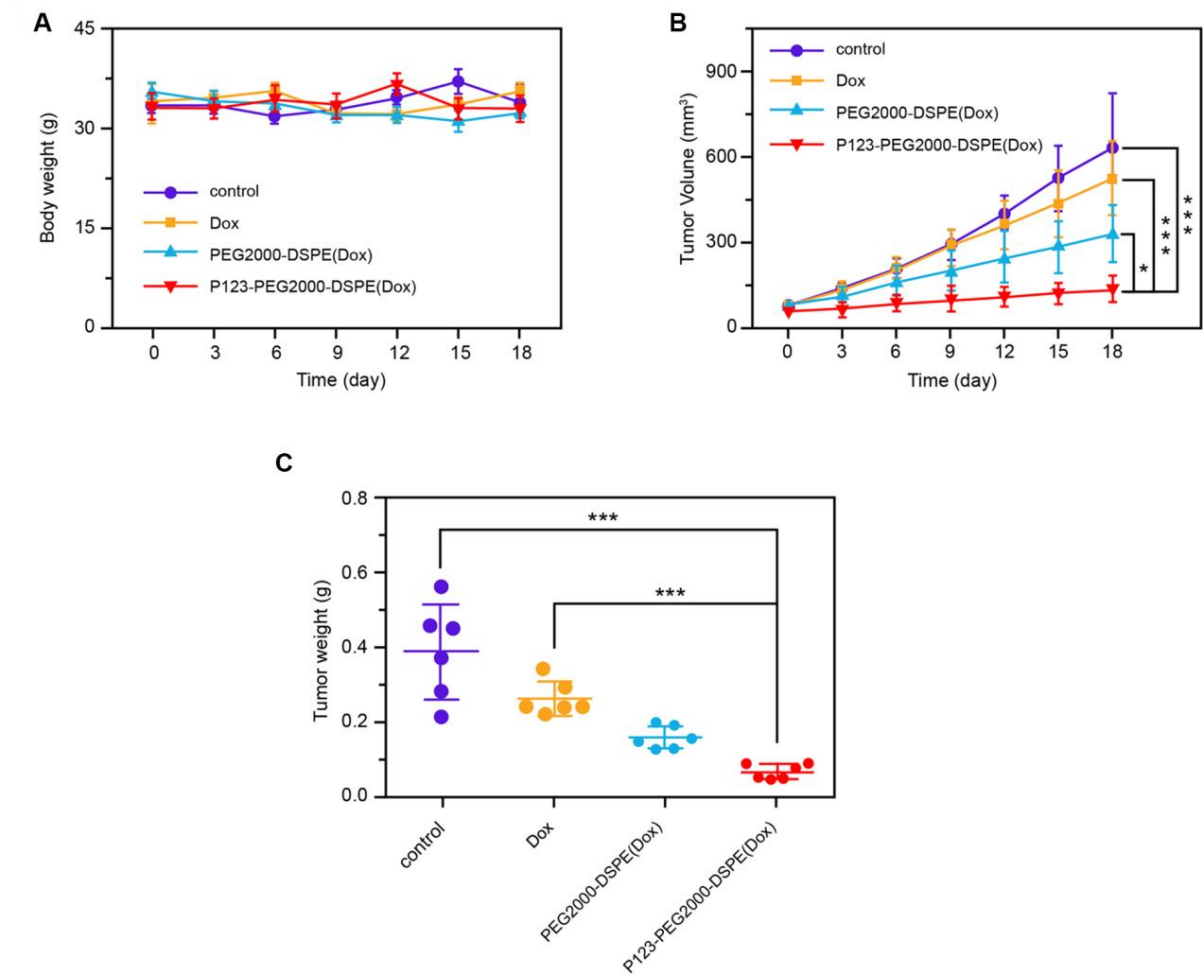
**P123-PEG2000-DSPE (Dox) exhibited better anti-tumor effect of on drug-resistant BC mice.** (**A**) The body weights of mice with different treatments, including saline solution (control), Dox, PEG2000-DSPE (Dox), and P123-PEG2000-DSPE (Dox), respectively, every three days for 18 days. (**B**) The tumor volumes of mice with different treatments, including saline solution (control), Dox, PEG2000-DSPE (Dox), and P123-PEG2000-DSPE (Dox), respectively, every three days for 18 days. (**C**) The tumor weights of mice with different treatments, including saline solution (control), Dox, PEG2000-DSPE (Dox), and P123-PEG2000-DSPE (Dox), respectively, on 18 days. ***P < 0.001

In vivo experiments showed that the body weights of mice with different treatments, including saline solution (control), Dox, PEG2000-DSPE (Dox), and P123-PEG2000-DSPE (Dox), were similar (Figure 7A). In addition, the tumor volume of mice was decreased after Dox treatment compared with control group in a time-dependent manner, and the tumor volume was further reduced in PEG2000-DSPE (Dox) and P123-PEG2000-DSPE (Dox) groups, especially in P123-PEG2000-DSPE (Dox) group (p < 0.05, Figure 7B). Moreover, compared to control groups, the tumor weight of mice was the lowest in the P123-PEG2000-DSPE (Dox) group, followed by PEG2000-DSPE (Dox) and Dox groups (p < 0.05, Figure 7C), which suggested the better anti-tumor effect of P123-PEG2000-DSPE (Dox) on drug-resistant BC mice.

## DISCUSSION

In the present study, we successfully prepared PEG2000-DSPE (Dox) and P123-PEG2000-DSPE (Dox) with a successful Dox release within 24 h. P123 modification significantly increased the cellular uptake of NMs and inhibited the expression of P-gp. Moreover, compared with free Dox, both PEG2000-DSPE (Dox) and P123-PEG2000-DSPE (Dox) significantly diminished cell viability and induced cell apoptosis in MCF-7R cells.

As one of the common chemotherapeutic drugs, Dox has been widely applied for the clinical treatments of acute leukemia, malignant lymphoma, BC, lung cancer, bladder cancer and other various cancers [16]. In this study, free Dox significantly inhibited cell viability and increased the rate of apoptotic cells in MCF7 cells and MCF7-R cells, which were supported by previous clinic trials. However, due to poor water solubility, many hydrophobic anti-cancer drugs are clinically limited to apply into cancer treatment [25]. NMs have been reported to be a promising drug delivery system to overcome the application limitation of hydrophobic drugs because of its attractive properties, including highly-hydrophilic nature and excellent biocompatibility [26, 27]. Greish K et al. [28] have reported that raloxifene-loaded NMs can improve cellular uptake and reduce tumor growth of triple-negative BC in vitro. Another *in vitro* study has also shown that quercetin loaded NMs significantly inhibit cell proliferation and induce apoptosis compared with free quercetin in PC-3 cells [29]. Moreover, *in vivo* experiments, due to increased accumulation and penetration of quercetin at the tumor site, quercetin loaded NMs also exert superior antitumor efficacy in the PC-3 xenograft mouse model [29]. Herein, Dox as a hydrophobic anti-cancer drug was loaded into the NMs in order to improve drug deliver. Consistent with previous studies, our study also revealed that compared with free Dox, both PEG2000-DSPE (Dox) and P123-PEG2000-DSPE (Dox) significantly diminished cell proliferation and induced cell apoptosis in MCF-7R cells. Interestingly, our study also found that higher dose (80%) of Dox released from both PEG2000-DSPE (Dox) and P123-PEG2000-DSPE (Dox) within 24 h at pH 5.0 (tumor endocytic compartment) than that at pH 7.4 (blood plasma), which indicated that more Dox was released under the tumor environment. These results confirmed the preferable anti-tumor effects of PEG2000-DSPE (Dox) and P123-PEG2000-DSPE (Dox) than free Dox *in vitro*.

Furthermore, due to failed chemotherapy caused by drug resistance to chemotherapeutic drugs, we focused whether P123 modified NMs could improve anti-tumor effects. It is well-known that P-gp as a drug transporter is highly expressed in MDR cancer cells, and involved in the alteration of drug pharmacokinetics by mediating drug metabolism, distribution, absorption, and excretion [30, 31]. Moreover, the overexpression of P-gp might increase the susceptibility of people to diseases [30, 31]. Importantly, increasing evidence has demonstrated that the effects of some drugs can be improved by modulating MDR1 gene and P-gp expression to increase the therapeutic effect of MDR tumors [11, 32, 33]. Recently, more attention has focused on P123 due to its biocompatible, safe, and relatively nontoxic properties, particularly, it can suppress the expression of P-gp [34]. Study has reported that lamotrigine loaded P123 NMs can overcome the activity of P-gp to enhance brain penetration of certain antiepileptic drugs via the blood-brain barrier [35]. Consistently, our study confirmed that P-gp was highly expressed in MDR MCF-7R cells but not MCF-7 cells. In addition, both P123-PEG2000-DSPE and P123-PEG2000-DSPE (Dox) could inhibit the expression of MDR1 gene and P-gp, and P123-PEG2000-DSPE (Dox) treatment further inhibited cell proliferation and promoted cell apoptosis in MCF-7 cells compared other PEG2000-DSPE (Dox). These results indicated that P123-PEG2000-DSPE (Dox) possessed enhanced anti-tumor effects than PEG2000-DSPE (Dox) and free Dox through inhibiting the expression of MDR1 gene and P-gp and then promoting drug excretion.

In conclusion, this study successfully developed P123-PEG2000-DSPE (Dox), and P123-PEG2000-DSPE (Dox) had a superior anti-tumor activity than PEG2000-DSPE (Dox) in MCF-7R cells by reversing MDR based on P-gp-mediated drug excretion and drug resistance mechanisms. Overall, P123-PEG2000-DSPE (Dox) might be a promising therapeutic nanomedicine in MDR BC.

## MATERIALS AND METHODS

### Preparation of NMs

In briefly, Dox hydrochloride was dissolved in 1 mL of absolute methanol, and then triethylamine (the mass ratio of Dox to triethylamine was 1:2) was added into the above solution to obtain hydrophobic Dox. Subsequently, hydrophobic Dox was mixed with PEG2000-DSPE that dissolved in 3 mL of chloroform at the mass ratio of 1:5, and then transferred into the eggplant-shaped bottle. After removed the organic solvent through a vacuum rotary evaporator, a dry lipid film was formed on the bottom of the bottle, and then dissolved in 2 mL of PBS buffer and hydrated in a water bath at 60°C for 30 min. The hydrated solution was filtered through polycarbonate membrane (0.2 μm of pore size) to remove unencapsulated hydrophobic Dox, thereby obtaining a Dox-loaded NMs (PEG2000-DSPE (Dox)) solution. Similarly, P123 and PEG2000-DSPE mixed Dox-loaded NMs (P123-PEG2000-DSPE(Dox)) were prepared as described above, except that PEG2000-DSPE was replaced by the mixture of Pluronic P123 and PEG2000-DSPE with a mass ratio of 1:4. Meanwhile, unloaded NMs (PEG2000-DSPE) were prepared as described above but without the addition of Dox.

### Determination of drug loading capacity (LC) and encapsulation efficiency (EE)

The concentration of Dox in P123-PEG2000-DSPE (Dox) was measured by a fluorescence spectrophotometer. Dox was set to have an excitation wavelength of 485 nm and an emission wavelength of 592 nm. The formulas for LC and EE of P123-PEG2000-DSPE (Dox) are as follows: EE% = Wt / Ws × 100% and LC% = Wt / Wo × 100%. Wt indicates the mass of Dox encapsulated in NMs; Wo: initial dose of Dox; Ws: total mass of NMs after lyophilization.

### Characterization of NMs

The morphological characteristics P123-PEG2000-DSPE (Dox) were observed by transmission electron microscope (TEM). Briefly, the NMs solution was diluted with deionized water at 0.25 mg/mL with deionized water, and then 10 μL of the sample was dripped onto a carbon-coated copper mesh. After water evaporation, the sample was counterstained with 5 μL of 1% uranyl acetate solution for 30 s, and dried by a 42°C constant temperature dryer. Ultimately, the sample was observed by TEM (Tecnai G2 20 S-TWIN, FEI, Eindhoven, Netherlands). The zeta potential and mean hydrodynamic size of P123-PEG2000-DSPE (Dox) were measured by dynamic light scattering (DLS) using Zetasizer Nano Z (Worcestershire, UK).

### In vitro drug release analysis

The drug release profiles of PEG2000-DSPE (Dox) and P123-PEG2000-DSPE (Dox) at different pH values were analyzed. In brief, 200 μL of PEG2000-DSPE (Dox) and P123-PEG2000-DSPE (Dox) were separately loaded into a dialysis bag (molecular retention of 8,000-12,000 Da), and the dialysis bag was immersed in 35 mL of PBS buffer (pH 7.4 and 5.0, respectively). PBS buffer with pH 7.4 simulated blood plasma environment and pH 5.0 simulated tumor endocytic compartment. The entire dialysis system was shaken at 200 rpm in a constant temperature shaker at 37°C in the dark. Subsequently, 1 mL of dialysate was taken at 0.5 h, 1 h, 2 h, 4 h, 6 h, 9 h, 12 h, and 24 h, respectively. Finally, the concentration of Dox in the dialysate was determined by fluorescence spectrophotometer, and the *in vitro* release profile was calculated.

### Cell culture and P-glycoprotein (P-gp) expression

Human BC cell line MCF-7 and the drug-resistant cell line MCF-7R were provided by Shanghai Obio (China), and maintained in RPMI-1640 medium (Gibco) with 10% fetal bovine serum (Gibco) under 37°C and 5% CO_2_. In addition, the expression of P-gp in MCF-7 and MCF-7R cells was detected.

### MTT assay

To evaluate the cytotoxicity of PEG2000-DSPE (unloaded NMs), MCF-7 and MCF-7R cells were cultured in 96-well plates. On the next day, cells were then incubated with PEG2000-DSPE at different concentrations (ranging from 0.05 to 500 μg/mL) of Dox, respectively, for 24 h. Next, MTT (10 μL, Sigma, St Louis, MI, USA) was added to incubate with cells for 4 h, and dimethyl sulfoxide (150 μL, Sigma) was then used to dissolve formazan precipitates. Microplate spectrophotometer was used to evaluate cell viability based on the absorbances at 450 nm. Moreover, to investigate the anti-tumor proliferation activity of NMs, MCF-7 and MCF-7R cells were incubated with Dox, PEG2000-DSPE (Dox) and P123-PEG2000-DSPE (Dox) at different concentrations (ranging from 0.01 to 100 μg/mL) of Dox, respectively, for 24 h, and the cell viability was evaluated by MTT assay.

### Cellular uptake of NMs in vitro

Cellular uptake of Dox, PEG2000-DSPE (Dox) and P123-PEG2000-DSPE (Dox) was evaluated using confocal imaging analysis. Briefly, MCF-7R cells (1.5 × 10^5^) were grown in 35 mm culture dishes for 24 h, and then incubated with Dox, PEG2000-DSPE (Dox) and P123-PEG2000-DSPE (Dox) at 10 μg/mL of Dox, respectively, for 0.5 h and 1 h. After washed with PBS, cells were observed using confocal fluorescence microscope with 488 nm of excitation wavelength.

### Cell apoptosis assay

FITC-Annexin V Apoptosis kit was used in this experiment. MCF-7R cells were treated with PBS (control), Dox, PEG2000-DSPE (Dox) and P123-PEG2000-DSPE (Dox), respectively, for 24 h. Next, cells were rinsed with PBS, and resuspended with Binding Buffer. After the incubation of FITC-Annexin V and PI with cells for 15 min, flow cytometer (BD, CA, USA) was used to calculate the number of apoptotic cells.

### qRT-PCR

Total RNA from untreated MCF-7 and MCF-7R cells as well as MCF-7R cells underwent various treatments was obtained by Trizol (Invitrogen), respectively, and then PrimeScript™ RT reagent Kit (Takara, Dalian, China) was utilized to obtain cDNA by reverse transcription of RNA. The qRT-PCR was carried out by the SYBR Premix Ex Taq TM II (Takara). The PCR primers for multi-drug resistance (MDR1) gene sense primer was 5'-ATATCAGCAGCCCACATCAT-3' and antisense primer was 5'-GAAGCACTGGGATGTCCGGT-3'; and glyceraldehyde-3-phosphate dehydrogenase (GAPDH) sense primer was 5'-GTGGATCAGCAAGCAGGAGT-3' and antisense primer was 5'-AAAGCCATGCCAATCTCATC-3'. GAPDH were served as the internal control, and mRNA expression data was evaluated by 2^-ΔΔCt^ method.

### Western blotting assay

Total proteins form untreated MCF-7 and MCF-7R cells as well as MCF-7R cells underwent various treatments were obtained using lysis buffer, respectively, and then quantitated by bicinchoninic acid kit (Beyotime, Shanghai, China). Following sample separating and transferring into PVDF membranes, membranes were immerged in 5% nonfat milk. Next, primary antibodies of P-gp (1: 800, Abcam), and β-actin (1: 1000, Beyotime), respectively, were used for immunoblotting of the membranes overnight at 4°C. After the incubation with second antibody (1:5000, Jackson, USA), the protein levels were detected by enhanced chemiluminescence (ECL, Millipore, USA).

### Cell immunofluorescence

Immunofluorescence assay was carried out as following. In brief, the cells with different treatments were gradually underwent paraformaldehyde and blockage. Then, the cells were reacted with P-gp antibody (1: 50, Abcam) overnight at 4°C, followed by incubation of the FITC-conjugated secondary antibody for 1 h. After washed with PBS, cells were observed by fluorescence microscope.

### Animal model and treatments

Approval from the local animal Ethics Committee of the animal laboratory center was obtained prior to experiments. Healthy female drug-resistant BC C57 mice (weighting 18-22 g, purchased from Charles River, Beijing, China) were used for the following experiments after one week of acclimation. Drug-resistant BC mice were treated with saline solution (control group, n = 8), Dox (10 mg/kg, Dox group, n = 8), PEG200-DSPE (Dox) (containing 10 mg/kg of Dox, PEG200-DSPE (Dox) group, n = 8), and P123-PEG200-DSPE (Dox) (containing 10 mg/kg of Dox, P123-PEG200-DSPE (Dox) group, n = 8) via rapid tail vein injection, respectively. The body weight, and tumor volume of mice were monitored every three days for 18 days. On day 18, mice were euthanized, and the tumor was resected for the measurement of tumor weight.

### Statistical analysis

Data were presented as the mean ± SD. One-way ANOVA followed by multiple comparison was used for data comparisons based on GraphPad Prism 6 software. P < 0.05 was considered significant.
